# Compensation to the Secretary

**Published:** 1867-06

**Authors:** 


### COMPENSATION TO THE SECRETARY.

On motion of Dr. Pullen, the Permanent Secretary was authorized to draw an order on the Treasurer, which the President shall sign, for such an amount of money as shall cover his annual expenses in attending the regular meetings of the Association.

